# Pathophysiological Role of Genetic Factors Associated With Gestational Diabetes Mellitus

**DOI:** 10.3389/fphys.2022.769924

**Published:** 2022-04-04

**Authors:** B. Ortega-Contreras, A. Armella, J. Appel, D. Mennickent, J. Araya, M. González, E. Castro, A. M. Obregón, L. Lamperti, J. Gutiérrez, E. Guzmán-Gutiérrez

**Affiliations:** ^1^ Pregnancy Diseases Laboratory, Department of Clinical Biochemistry and Immunology, Faculty of Pharmacy, Universidad de Concepción, Concepción, Chile; ^2^ Department of Instrumental Analysis, Faculty of Pharmacy, Universidad de Concepción, Concepción, Chile; ^3^ Department of Obstetrics and Gynecology, Faculty of Medicine, Universidad de Concepción, Concepción, Chile; ^4^ Departamento de Obstetricia y Puericultura, Facultad de Ciencias de la Salud, Universidad de Atacama, Copiapó, Chile; ^5^ Faculty of Health Care, Universidad San Sebastián, Concepción, Chile; ^6^ Faculty of Health Sciences, Universidad San Sebastián, Santiago,Chile

**Keywords:** gestational diabetes mellitus, single nucleotide polymorphism, insulin resistance, genetic risk factors, insulin signaling dysfunction

## Abstract

Gestational Diabetes Mellitus (GDM) is a highly prevalent maternal pathology characterized by maternal glucose intolerance during pregnancy that is, associated with severe complications for both mother and offspring. Several risk factors have been related to GDM; one of the most important among them is genetic predisposition. Numerous single nucleotide polymorphisms (SNPs) in genes that act at different levels on various tissues, could cause changes in the expression levels and activity of proteins, which result in glucose and insulin metabolism dysfunction. In this review, we describe various SNPs; which according to literature, increase the risk of developing GDM. These SNPs include: (1) those associated with transcription factors that regulate insulin production and excretion, such as rs7903146 (*TCF7L2*) and rs5015480 (*HHEX*); (2) others that cause a decrease in protective hormones against insulin resistance such as rs2241766 (*ADIPOQ*) and rs6257 (*SHBG*); (3) SNPs that cause modifications in membrane proteins, generating dysfunction in insulin signaling or cell transport in the case of rs5443 (*GNB3*) and rs2237892 (*KCNQ1*); (4) those associated with enzymes such as rs225014 (*DIO2*) and rs9939609 (*FTO*) which cause an impaired metabolism, resulting in an insulin resistance state; and (5) other polymorphisms, those are associated with growth factors such as rs2146323 (*VEGFA*) and rs755622 (*MIF*) which could cause changes in the expression levels of these proteins, producing endothelial dysfunction and an increase of pro-inflammatory cytokines, characteristic on GDM. While the pathophysiological mechanism is unclear, this review describes various potential effects of these polymorphisms on the predisposition to develop GDM.

## Highlights

1) Several SNPs cause predisposition to GDM.
2) SNPs associated with GDM mainly affect endocrine pancreas function and adipose tissue response to insulin.
3) Physiopathology induced by these SNPs could explain GDM development.

## 1 Introduction

The American Diabetes Association (ADA) defines gestational diabetes mellitus (GDM) as a type of diabetes diagnosed at the second or third trimester of pregnancy in a mother not diagnosed with pregestational diabetes (ADA, 2020). In recent years, it has been observed that the incidence of this pathology is increasing along with obesity and type 2 diabetes mellitus (T2DM) (Coustan, 2013; ADA, 2019). It is estimated that the worldwide prevalence of GDM varies between 1.7 and 11.7% (Griffin et al., 2000; Schneider et al., 2012). This considerable variation is due to differences among the populations and diagnostic criteria used in each country. It has been estimated that countries with higher incidence of GDM are those of Middle East and North Africa with 12.9%, followed by Southeast Asia (11.7%) (Zhu and Zhang, 2016). In developed countries such as the United States, Australia, Canada and the United Kingdom, its prevalence is less than 6% (Zhao et al., 2016). South American countries show high GDM prevalence: about 15% of pregnant women from Peru and Chile have been diagnosed with GDM in the last 20 years (Belmar et al., 2004; Huidobro et al., 2004; Larrabure-Torrealva et al., 2018; Garmendia et al., 2019).

One of the proposed risk factors for GDM is obesity. In fact, GDM women usually have a body mass index (BMI) higher or equal to 25 kg/m^2^ (Shah et al., 2011). An increase in proinflammatory cytokines have been reported in obese pregnant women affected with GDM (Kinalski et al., 2005; Kuzmicki et al., 2008; Pantham et al., 2015). This pro-inflammatory state stimulates the synthesis of xanthurenic acid, which has been associated with the development of T2DM, prediabetes, and GDM (Bennink and Schreurs, 1975; Oxenkrug, 2015; Law and Zhang, 2017). Moreover, in GDM pregnant women there is a supraphysiological insulin resistance state induced in part by pro-inflammatory cytokines (Sonagra et al., 2014). For that reason, GDM pregnancies are associated with high HOMA-IR index values (Wang et al., 2018).

The main maternal outcomes of GDM are hyperglycemia, GDM in future pregnancies, future development of T2DM (Griffin et al., 2000; Ben-Haroush et al., 2004), obesity, and preeclampsia (Coustan, 2013; Kampmann et al., 2015). A study by the HAPO Study Cooperative Research Group demonstrated that in GDM pregnancies, the main fetal outcomes are macrosomia, neonatal hyperinsulinemia, caesarean section, and neonatal hypoglycemia (HAPO Study Cooperative Research Group, 2002; HAPO Study Cooperative Research Group, 2008). Furthermore, newborns from GDM pregnancies have a high risk of developing T2DM and obesity in the long-term (Dabelea et al., 2000).

Maternal and fetal outcomes are associated with modifiable and non-modifiable factors (Shaat et al., 2007). One of these non-modifiable factors is genetics. In this line, diverse Genome-Wide Association Studies (GWAS) have shown that genetic variables of the single nucleotide polymorphisms (SNPs) type associated with T2DM have also been related with high predisposition to GDM, which has been studied in diverse populations (Kwak et al., 2012; Huerta-Chagoya et al., 2015; Lowe et al., 2016). This association has been proposed because both GDM and T2DM are associated with similar pathophysiology mechanisms, including insulin resistance and a chronic inflammatory state (Zajdenverg and Negrato, 2017). Furthermore, GDM increases the risk of progression to T2DM; nevertheless, the magnitude of this effect is variable in different populations (Plows et al., 2018; Vounzoulaki et al., 2020). Unlike T2DM, GDM is triggered by placental and maternal hormones that cause a transitory insulin resistance that in most cases disappears after pregnancy, and only affects pregnant women (Mack and Tomich, 2017).

The positive associations of GDM and genetic variations is not clear in all cases (Anghebem-Oliveira et al., 2017), and the mechanisms by which these could contribute to GDM development have not been fully described. Therefore, this review summarizes the main genetic variants that have been described for GDM, emphasizing their potential pathophysiological mechanisms on the generation of GDM.

## 2 Genetic Factors Associated With Gestational Diabetes Mellitus and Its Pathophysiological Mechanism Over the GDM Etiology

GDM etiology could be associated with genetic variations that are related to T2DM development (Buchanan and Xiang, 2005; Gong et al., 2016). GWAS have identified numerous loci associated with the risk of GDM (Dalfrà et al., 2020). In this review, we classify different SNPs that have been associated with T2DM and GDM, according to the protein they encode (Table 1).

**TABLE 1:** Single nucleotide polymorphisms associated with gestational diabetes mellitus.

Gene	SNP	Number of Participants	Population	Genetic Variant	OR (95%CI)	References
*ADIPOQ*	rs2241766	135 controls and 135 GDM	Chinese	TG + GG vs. TT	1.67 (1.03–2.70)	Feng et al. (2019)
				G allele	1.55 (1.08–2.23)	
*CDKAL1*	rs7754840	2025 controls and 1399 GDM	Korean	C allele	1.52 (1.37–1.68)	Kwak et al. (2012)
	rs7748720	315 controls and 319 GDM	Chinese	AA+ GA vs. GG	1.46 (1.01–2.10)	Wang et al. (2019)
	rs6938256			GG + AG vs. AA	0.58 (0.42–0.81)	
*DIO2*	rs225014	516 controls and 1057 T2DM	Brazilian	C allele	1.18 (1.03–1.36)	Dora et al. (2010)
*FTO*	rs9939609	7229 controls and 3636 GDM	Multi-ethnic (Meta-analysis)	AA vs. TT	1.33 (1.05–1.68)	Lin et al. (2018)
				AA vs. AT + TT	1.31 (1.07–1.61)	
				A vs. T	1.12 (1.01–1.28)	
	rs1121980	1021 controls and 964 GDM	Chinese	A allele	0.79 (0.67–0.94)	Cao et al. (2020)
*GLIS3*	rs10814916	6086 controls and 2636 GDM	American and Danish	C allele	1.16 (1.08–1.24)	Ding et al. (2018)
	rs7041847			A allele	1.13 (1.05–1.20)	
*GNB3*	rs5443	130 controls and 120 GDM	Chinese	CT + TT vs. CC	1.91 (1.05–3.46)	Feng et al. (2019)
*GPSM1*	rs11787792	6086 controls and 2636 GDM	American and Danish	A allele	0.87 (0.80–0.94)	Ding et al. (2018)
*HHEX*	rs5015480	204 GDM and 207 NGT	Polish	C allele	1.40 (1.05–1.87)	Tarnowski et al. (2017)
		18 studies (18227 GDM and 30366 controls)	Multi-ethnic (Meta-analysis)		1.16 (1.06–1.26)	Li et al. (2012)
		4 studies (3513 controls and 1651 GDM)	Multi-ethnic (Meta-analysis)		1.24 (1.12–1.38)	Wang et al. (2020)
*HNF1A*	rs7957197	6086 controls and 2636 GDM	American and Danish	T allele	1.22 (1.12–1.33)	Ding et al. (2018)
*KCNQ1*	rs2237892	453 controls and 562 GDM	Chinese	C allele	2.19 (1.36–3.54)	Ao et al. (2015)
	rs163182	1021 controls and 964 GDM	Chinese	C allele	0.84 (0.73–0.96)	Cao et al. (2020)
*MC4R*	rs12970134	1021 controls and 964 GDM	Chinese	A allele	1.25 (1.07–1.46)	Cao et al. (2020)
	rs2229616	676 controls and 753 GDM	Chinese	T allele	1.62 (1.05–2.50)	Shen et al. (2020)
*MIF*	rs755622	485 controls and 430 GDM	Chinese	C allele	1.59 (1.28–1.98)	Li et al. (2016)
*MTNR1B*	rs10830962	2025 controls and 1399 GDM	Korean	G allele	1.45 (1.32–1.61)	Kwak et al. (2012)
	rs10830963	6086 controls and 2636 GDM	American and Danish	G allele	1.27 (1.18–1.37)	Ding et al. (2018)
	rs1387153			T allele	1.17 (1.09–1.26)	
	rs10830963	676 controls and 753 GDM	Chinese	G allele	1.36 (1.17–1.59)	Shen et al. (2020)
	rs1387153			T allele	1.40 (1.20–1.63)	
	rs1447352			G allele	0.82 (0.69–0.97)	
	rs2166706			C allele	1.36 (1.17–1.59)	
	rs4753426			T allele	0.84 (0.71–0.99)	
*PROX1*	rs340841	1021 controls and 964 GDM	Chinese	T allele	1.22 (1.07–1.39)	Cao et al. (2020)
*RREB1*	rs9379084	6086 controls and 2636 GDM	American and Danish	A allele	0.80 (0.71–0.90)	Ding et al. (2018)
*SLC30A8*	rs3802177	6086 controls and 2636 GDM	American and Danish	G allele	1.17 (1.08–1.26)	Ding et al. (2018)
*SHBG*	rs6257	359 controls and 359 T2DM	American	C allele	1.68 (1.07–2.64)	Ding et al. (2009)
*TCF7L2*	rs7903146	810 controls and 210 GDM	German	T allele	1.52 (1.11–2.069)	Fritsche et al. (2018)
		6086 controls and 2636 GDM	American and Danish		1.15 (1.06–1.24)	Ding et al. (2018)
		5639 controls and 1422 T2DM	Swedish	CT + TT vs. CC	1.58 (1.38–1.81)	Lyssenko et al. (2007)
		2501 controls and 150 T2DM	Finish		1.61 (1.14–2.27)	
		6473 controls and 3404 T2DM	Multi-ethnic (Meta-analysis)	TT vs. TC + CC	1.65 (1.42–1.65)	Liu et al. (2015)
				T allele	1.53 (1.35–1.72)	
	rs34872471	6086 controls and 2636 GDM	American and Danish	G allele	1.14 (1.06–1.23)	Ding et al. (2018)
	rs4506565	6086 controls and 2636 GDM	American and Danish	T allele	1.16 (1.08–1.24)	Ding et al. (2018)
	rs12255372	5639 controls and 1422 T2DM	Swedish	GT + TT vs. GG	1.42 (1.24–1.62)	Lyssenko et al. (2007)
*TNF-α*	rs1800629	181 controls and 196 GDM	Multi-ethnic (Meta-analysis)	A allele	1.38 (0.37–5.16)	Wu et al. (2016)
*VEGF*	rs2146323	275 controls and 239 GDM	Chinese	AA vs. CC	2.00 (1.04–3.86)	Dong, (2019)
				CA + AA vs. CC	1.49 (1.05–2.13)	
				A allele	1.46 (1.10–1.94)	
	rs3025039			CT vs. CC	1.95 (1.36–2.80)	
				TT vs. CC	6.03 (1.95–18.65)	
				CT + TT vs. CC	2.12 (1.49–3.02)	
				T allele	1.89 (1.42–2.23)	

SNP, single nucleotide polymorphism; OR, odd ratio; CI, confidence interval; GDM, gestational diabetes mellitus; T2DM, type 2 diabetes mellitus.

The presence of genetic variants can result in changes in the expression and function of the encoded protein, affecting diverse physiological actions. For this reason, polymorphisms can be clinically relevant for various pathologies (Ramírez-Bello and Jiménez-Morales, 2017). In the following section, we focus on explaining the pathophysiological mechanisms caused by the main aforementioned genetic factors.

### 2.1 Polymorphisms Associated With Transcription Factors

#### 2.1.1 *TCF7L2* Genetic Variants Associated to GDM

The transcription factor 7-like 2 (*TCF7L2*) gene is located on chromosome 10 and encodes for a nuclear protein that participates in gene expression regulation involved in the fusion of insulin secreting granules in pancreatic beta cells (da Silva et al., 2009). In a multiracial study, carriers of the T allele of SNP rs7903146 (risk-allele) showed an increased risk of T2DM (OR: 1.58) and a faster deterioration of insulin secretion (Lyssenko et al., 2007; Zhang et al., 2013; Liu et al., 2015). Interestingly, Asian pregnant women homozygous for the TT genotype, had a higher risk of developing GDM (OR = 2.08), followed by Hispanic/Latin (OR = 1.80), and white (OR = 1.51) (Lin et al., 2016). Other ethnic groups have also been studied (Shaat et al., 2007; Zhang et al., 2013; Wu et al., 2016). Therefore, the presence of the T allele in the rs7903146 variant could be a genetic risk factor for GDM. Other SNPs of the same gene have been related with a higher risk to develop GDM in a meta-analysis (Chang et al., 2017), i.e., carriers of the T allele for the rs12255372 variant (OR: 1.46) (Zhang et al., 2013).

TCF7L2 is expressed in several tissues, including the islets of Langerhans, and liver (Zhou et al., 2014). This factor participates as a transcriptional effector in the Wnt signaling pathway where it regulates the transcription of diverse genes, including some of those involved in the production and function of incretin hormones and in blood glucose homoeostasis (Schinner et al., 2008; Ip et al., 2012).

Reduced mRNA levels of this gene in the pancreatic islets are related to a significant increase in the apoptosis of beta cells and a decrease in their proliferation, which causes a reduction in insulin secretion (Shu et al., 2008; Savic et al., 2011). Moreover, evidence in the murine model *Tcf7l2* (null), shown an alteration in glucose metabolism causing hypoglycemia. In contrast, the overexpression of this gene in the same animal model using *Cre* recombinase resulted in glucose intolerance (Savic et al., 2011). This results are related to the high expression levels of *TCF7L2* mRNA in T2DM patients (Lyssenko et al., 2007), and could be an interesting idea for study in GDM models.

The SNP rs7903146 of the *TCF7L2* gene is located in its intronic region upstream exon 5 in a regulatory site, specifically in an islet-selective open chromatin site. This means that in human islet cells, the chromatin state at rs7903146 is more open in chromosomes carrying the T allele, and have a greater enhancer activity compared to the C allele (Gaulton et al., 2010). This could explain the association of the T-allele with an impaired glucose-stimulated insulin secretion (Dahlgren et al., 2007). Moreover, carriers of this risk allele exhibit a significantly higher expression of *TCF7L2* mRNA in the pancreatic islets and an increased hepatic glucose production (Lyssenko et al., 2007). Carriers of the TT genotype for this polymorphism have higher concentrations of blood glucose, proinsulin, and incretin hormones, compared to the normal genotype group (Gjesing et al., 2011). Likewise, the insulinogenic index, derived from an oral glucose tolerance test, is diminished in carriers of the T-allele, which would be associated with an impaired insulin secretion and not with resistance to this hormone (Saxena et al., 2006). However, there are not work in GDM models, or human that demonstrated this association in pregnancies with glucose intolerance.

#### 2.1.2 *HHEX* Genetic Variant Associated to GDM

GWAS have associated several polymorphisms with GDM, and the Hematopoietically-expressed homeobox (*HHEX*) gene rs5015480 stands out among them. This gene codifies for a transcription factor that is part of a homeobox gene family involved in developmental and hematopoietic differentiation processes (Bedford et al., 1993). In a study conducted in Poland, the risk C-allele was associated with a genetic predisposition to develop GDM (OR: 1.40) and also to an increased BMI in pregnant women (Tarnowski et al., 2017). In addition, a meta-analysis showed a strong association of the CC genotype in contrast to the TT genotype (OR: 1.65) in different populations (Wang et al., 2020).

HHEX is a transcription factor that is also linked to the Wnt signaling pathway. It is essential for cell growth and for the development of organs such as the thyroid, pancreas, liver, and brain (Tarnowski et al., 2017). In the adult endocrine pancreas, this factor is selectively expressed in the somatostatin-secreting delta cells, where it has been observed to regulate the differentiation of this cell type (Zhang J. et al., 2014). It is described that a decrease in somatostatin levels causes a paracrine inhibition of the insulin release from β cells (Moldovan et al., 1995). In this context, it has been suggested that this transcription factor directly activates the transcription of the somatostatin gene (Zhang J. et al., 2014).

In the *HHEX* gene, the risk C-allele of the rs5015480 variation is associated with altered β cells secretion. Other variations of this gene are also involved in reduced fasting insulin secretion, insulin sensitivity, and glucose-stimulated insulin secretion (Pivovarova et al., 2009). Some authors have proposed that the rs5015480 variation could affect early stages of insulin secretion due to the reduced insulinogenic index, described in risk allele carriers (Dimas et al., 2014), which could be related to insulin secretion regulation exerted by delta cells (Zhang J. et al., 2014).

Genetic modifications in these transcription factors could cause alterations in the protein expression involved in the development of GDM. In fact, it is proposed that both TCF7L2 and HHEX have an important role in the regulation of insulin secretion in GDM (Saxena et al., 2006; Dimas et al., 2014). In fact, the polymorphisms *TCF7L2* rs7903146 and *HHEX* rs5015480 could decrease insulin secretion during pregnancy, favoring GDM development due to an alteration at the level of pancreatic cells; and consequently, in the production and secretion of insulin.

### 2.2 Polymorphisms Associated With Hormones

#### 2.2.1 *ADIPOQ* Genetic Variant Associated to GDM

A relevant hormone in GDM is adiponectin, due to its protective role against insulin resistance. The risk G-allele in rs2241766 (T > G) produces a silent mutation in nucleotide 45 in the adiponectin gene (*ADIPOQ*). This genetic variation is associated with obesity in several studies. Also, carriers of the G/G genotype for this polymorphism had a higher risk of T2DM (OR: 1.70) compared to the T/T genotype (Hara et al., 2002). Moreover, a meta-analysis (Bai et al., 2020) demonstrated an increased risk of GDM in Asian (OR: 2.08) and European (OR: 1.52), but a diminished risk in the American population (OR: 0.642) carriers of the G allele of this SNP.

The adiponectin hormone is synthesized in adipose tissue, where it modulates diverse metabolic processes, among them: metabolism of lipids and fatty acids, reduction of plasmatic triglycerides and improvement of glucose metabolism by an increase in insulin sensitivity (Karbowska and Kochan, 2006). Moreover, adiponectin reduces the expression of adhesion molecules in endothelial cells, the transformation of macrophages into foamy cells, the expression of tumor necrosis factor α (TNF-α) and the proliferation of smooth muscle tissue cells (Mierzyński et al., 2018).

There are genetic variations in the adiponectin gene (*ADIPOQ*) such as rs2241766, where the risk G-allele produces a silent mutation in nucleotide 45, which does not cause an amino acid change. However, being very close to the exon-intron limit, it could affect the splicing machinery (Salazar et al., 2010). The presence of this allele can completely inactivate the activity of the adiponectin promoter and the expression of the *ADIPOQ* gene, and thus can decrease adiponectin levels (Chu et al., 2013).

During pregnancy, the plasmatic levels of this hormone are normally decreased; however, such levels are even lower in the presence of this variation (Huang et al., 2019). In fact, lower levels of adiponectin could be related to an increase in the formation of dense LDL particles (Lara-Castro et al., 2007), and to an increase in the expression of pro-inflammatory cytokines such as TNF-α, IL-6 and IL-8 (Hussain et al., 2018).

All this evidence supports the idea that the adiponectin gene could be a susceptibility factor for developing GDM (Takhshid et al., 2015; Xu et al., 2016).

#### 2.2.2 *SHBG* Genetic Variant Associated to GDM

The *SHBG* gene codifies for the sex hormone-binding globulin, which could contribute to the pathophysiology of GDM. This gene has been associated with the risk of diabetes mellitus (Ding et al., 2009; Hedderson et al., 2014). In fact, low levels of pre-gestational SHBG increase the risk of developing GDM (OR: 4.06) (Hedderson et al., 2014).

In addition, the rs6257 (T > C) polymorphism of this gene has been associated with plasmatic concentrations of SHBG. It has been shown that carriers of the CC or CT risk genotypes have lower plasmatic concentrations of this hormone compared to the carriers of the normal TT genotype (Ding et al., 2009; Hedderson et al., 2014). Although there are still not studies that demonstrate association between GDM and these genetics variants, changes in SHBG concentration due to these variants, could increase this risk.

SHBG is synthesized in the liver and is responsible for the transport of androgens in circulation and for the regulation of the bioavailability of these hormones. It is also involved in receptor-mediated processes where it regulates the effects of dihydrotestosterone (DHT) and estradiol (Fortunati, 1999).

An *in-silico* study postulates that rs6257 favors the union of the *forkhead box protein A2* (FOXA2) element to the *SHBG* gene, repressing its transcription by a splicing defect (Ding et al., 2009). This idea was tested in HepG2 cells (Wu, 2015). Furthermore, it is described that in GDM there is a decrease in the plasmatic concentrations of SHBG compared to pregnant women without this pathology (Bartha et al., 2000; Tawfeek et al., 2017; Faal et al., 2019). This phenomenon was also studied in a trophoblast cell model (HTR8 Sv-neo) exposed to high levels of insulin, showing a decrease in SHBG mRNA and protein levels (Zhang et al., 2016; Feng et al., 2018). This could be explained by a reduction in the signaling of the phosphatidylinositol 3-kinase (PI3K/Akt) pathway, which mediates the transduction of insulin signals (Feng et al., 2018). Although literature is scarce, the reduction of GLUT-4, GLUT-3 and IRS-1 expression in GDM patients could be correlated with a lower SHBG activity, which could favor insulin resistance (Zhang et al., 2016).

### 2.3 Polymorphisms Associated With Membrane Proteins

#### 2.3.1 *GNB3* Genetic Variant Associated to GDM

Heterotrimeric G-proteins are relevant components of transmembrane receptors and are involved in the regulation of different intracellular signaling pathways. The 825C > T SNP in the gene of the G-protein β3 subunit (*GNB3*) has been linked to metabolic features such as hypertension, atherosclerosis and immunological response. The T-allele has also been associated with obesity risk in German, Chinese and South African populations (Siffert et al., 1999). In pregnant women, carriers of the risk allele have a higher weight gain during gestation (Dishy et al., 2003). Interestingly, it has also been described that the CT and TT genotypes are significantly related to a higher risk of GDM (OR: 1.91; 95%CI: 1.053–3.463) (Feng et al., 2019); however, there is still a lack of studies to confirm this relationship.

The G protein is involved in the regulation of glucose levels through the metabolic pathway of insulin signaling. Also, it is involved in the stimulation of second messengers such as adenylate cyclase, epinephrine signaling pathways and glucagon receptors in the liver, muscular and fatty tissue cells (Rizvi et al., 2016).

The most studied polymorphism of the *GNB3* gene is C825T, which is produced in exon 10 and it generates an alternative splicing causing the loss of 41 amino acids, structurally modifying this protein. The risk T-allele has been associated with a higher production of the G protein beta three subunit, causing an increased activation of this protein. With that increased activation comes an enhanced activity of the potassium channels at the cardiac level and vasoconstriction mediated by α-adrenoreceptors, which are directly related with arterial hypertension (Andersen et al., 2006). Another study demonstrated that the CC genotype of this SNP might be associated with higher obesity-related metabolic traits, such as triglyceride and total cholesterol in non-obese subjects (Hsiao et al., 2013).

Unfortunately, there is no direct mechanism that explains the relationship of this polymorphism and insulin resistance. However, obesity-related metabolic traits are tightly linked to GDM pathophysiology.

#### 2.3.2 *KCNQ1* Genetic Variant Associated to GDM

It is suggested that the gene of the potassium voltage-gated channel subfamily Q member 1 (*KCNQ1*)*,* participates on the regulation of insulin secretion in the pancreas, and it has been described as one of the candidate genes for GDM. Evidence shows that the rs2237892 variation of this gene is significantly associated with increased glucose levels, impaired insulin secretion, and higher GDM risk (OR: 1.99) in Asian population (Yasuda et al., 2008; Shin et al., 2010; Ao et al., 2015). Correspondingly, a study including 637 Pakistani women associated the presence of the risk allele A of this polymorphism, with an enhanced GDM risk (OR: 2.07) (Fatima et al., 2016). On the other hand, the risk genotype of rs163182 has been associated with lower GDM risk (OR: 0.84) (Cao et al., 2020).

The *KCNQ1* gene encodes for the alpha subunit of the pore-forming potassium channel (KvLQT1) performing an important role in the control of the vascular repolarization process (Yasuda et al., 2008). This gene is expressed in epithelial cells, including those of the endocrine and exocrine pancreas. In addition, KCNQ1 channels are expressed in insulin-secretor INS-1 cells, where they depolarize the membrane potential of the pancreatic beta cells allowing insulin secretion (Kwak et al., 2010).

Genetic variations in the sequence of *KCNQ1* such as rs2237892 cause changes in the translation and/or in post-translational modifications, reducing β cells function (Jonsson et al., 2009). Thus, a decreased secretory capacity of the β cells could increase GDM risk by reducing the secretory capacity of insulin and limiting its compensation (Wang et al., 2013). In fact, GDM patients carrying the risk allele have higher fasting plasma glucose levels and lower insulin secretion (Wang et al., 2013).

### 2.4 Polymorphisms Associated to Enzymes

#### 2.4.1 *DIO2* Genetic Variant Associated to GDM

The *DIO2* gene codifies to an enzyme that is ubiquitous in the human body, and that regulates the conversion of tetraiodothyronine (T4) to triiodothyronine (T3). It is proposed that the rs225014 (T > C) polymorphism causes a change in the amino acid sequence from a threonine (Thr) to an alanine (Ala) on codon 92 (Thr92Ala) (Estivalet et al., 2011). This variation has been associated with higher GDM risk (OR: 1.29) (Asadi et al., 2016).

Deiodinase 2 (DIO2) beside the essential role in thyroid hormones homeostasis, is also involved in brain growth and maturation, glucose uptake in the muscle, among other effects (Loubiere et al., 2010; Galton et al., 2014; Yang et al., 2016). This enzyme is mainly found in the central nervous system, hypophysis, skeletal muscle, thyroid, heart, bones, and adipose tissue (Coppotelli et al., 2006).

The rs225014 genotype is associated with *DIO2* expression (Bomer et al., 2015), and the presence of the risk C allele, which causes the substitution of a Threonine for an Alanine, reducing the DIO2 activity (Estivalet et al., 2011). In fact, it has been observed that homozygous patients for this polymorphism have a lower enzymatic activity, evidenced by a 37% decrease in DIO2 speed in skeletal muscle, and a reduction around 90% in its maximal velocity (*V*_max_). These results may explain the association of this variation with insulin resistance. As the skeletal muscle is the main site of insulin-dependent glucose uptake, a lower DIO2 activity would decrease the amount of T3 generated in the skeletal muscle, and with this, the expression of genes involved in energy use, such as GLUT4, leading to insulin resistance (Canani et al., 2005).

#### 2.4.2 *FTO* Genetic Variant Associated to GDM

The fat mass and obesity-associated gene (*FTO*) codifies for a dioxygenase enzyme that is found within the cell nucleus. It is reported that *FTO* participates in mRNA processing and splicing processes. Regarding the *FTO* gene, the polymorphisms rs9939609, rs8050136, and rs1421085 are associated with BMI increase, risk of obesity, and T2DM. However, controversy still exists about the association between certain polymorphisms and GDM (Hotta et al., 2008; He et al., 2018). Indeed, rs8050136 and rs14211085 SNPs were not associated with GDM risk (de Melo et al., 2015; Anghebem-Oliveira et al., 2017; Lin et al., 2018; Tarnowski et al., 2018), but with proinflammatory state and weight gain during pregnancy (Saucedo et al., 2017).

The FTO rs9939609 (T > A) polymorphism is located in the first intron of this gene (Frayling et al., 2007). Recent evidence suggests that this polymorphism is strongly correlated with GDM risk (OR: 1.31) in Caucasian subjects (Lin et al., 2018). Also, the A-allele is associated with rapid weight gain during pregnancy (Lawlor et al., 2011; Martins et al., 2016).

These genetic variants could alter the expression or enzymatic activity of FTO, leading to changes in the metabolism that could impair glucose metabolism and generate insulin resistance (Saber-Ayad et al., 2019). Subsequently, the GDM risk could be increased.

The FTO protein acts within the nucleus demethylating the N6-methyladenosines in mRNA and regulating the splicing of genes involved in adipogenesis such as *FABP4*, *PPARγ*, *C/EBPα* and *PLIN1* (Zhao et al., 2014a; Ben-Haim et al., 2015; Merkestein et al., 2015; Zhang et al., 2015; Bartosovic et al., 2017).

In murine models, the *Fto* gene is widely expressed in the brain, including the hypothalamic nucleus, which is related to energy intake regulation (Speakman, 2015). Studies in primary culture of Mouse Embryonic Fibroblasts (MEFs) have demonstrated that FTO regulates the splicing of the *RUNX1T1* (Runt-related transcription factor 1) gene (Zhao et al., 2014b)*,* which is involved in the early stages of adipogenesis (Rochford et al., 2004). FTO exerts its action during the splicing of the mRNA transcript of this gene, causing the exclusion of exon 6 and generating a short pro-adipogenic isoform of RUNX1T1, which increases adipocytes proliferation. The latter is a condition that is favored when *FTO* is overexpressed (Merkestein et al., 2015). Moreover, *FTO* overexpression in C57B/6J mice is associated with weight gain and increased adipogenic activity (Merkestein et al., 2015).

Variations in the first intron of the FTO gene have been associated with higher BMI and T2DM, and it has been reported that there is a 47 kb region that comprehends several SNPs associated with these pathologies. The rs9939609 variant has been extensively studied (Frayling et al., 2007). The mechanism by which this variant causes obesity is still unclear, however, heterozygous subjects for this polymorphism have higher levels of primary FTO transcripts of the risk A-allele, than of the T-allele (Berulava and Horsthemke, 2010), and this could cause higher levels of FTO expression favoring adipogenesis. However, the latter relationship has not yet been reported in literature.

Another SNP close to rs9939609 and associated with an increased BMI is rs8050136. The DNA sequence that included A-allele of this polymorphism preferentially binds with CUTL1, a transcription factor that increases the FTO expression. It has been proposed that, given the proximity of these variants, the rs9939609 SNP could also increase FTO expression by the same mechanism (Stratigopoulos et al., 2008).

### 2.5 Polymorphisms Associated With Growth Factors

#### 2.5.1 *VEGFA* Genetic Variant Associated to GDM

Another group of genes associated with GDM are growth factors. One of them is the vascular endothelial growth factor A (*VEGFA*), which has an essential role in angiogenesis by inducing the migration of endothelial precursor cells from the bone marrow, and causing differentiation and proliferation of endothelial progenitor cells in angiogenesis sites (Saavedra et al., 2017). A study involving the *VEGFA* gene showed that the A allele of the rs2146323 polymorphism and the T allele of the rs3025039 variant, increase the risk of developing GDM (OR: 1.456 and 1.894, respectively) (Dong, 2019). Additionally, the frequency of the risk haplotypes of rs2010963, rs833069, rs2146323 and rs3025010 is higher in GDM patients compared to normal pregnancies (Dong, 2019).

VEGFA has the function of promoting endothelial cell proliferation and increasing vascular permeability to induce angiogenesis. In fact, the placentas of GDM patients show hypervascularization, which is explained by a greater demand of oxygen by the fetus due to an increase in fetal aerobic metabolism stimulated by insulin (Troncoso et al., 2017). A study conducted in a murine model with GDM induced by a high fat diet, showed that those who had GDM had a high placental inflammatory response evidenced by increased IL-1β and TNFα, and a higher degree of placental hypoxia denoted by an enhanced expression of inducible hypoxia factor-1α (HIF-1α) and VEGF-A. In this sample, altered placental vascular development due to hypervascularization was also present (Li, 2013). Therefore, in GDM, VEGF would be involved in angiogenesis processes in the placenta, but it is not clear whether genetic alterations could explain this mechanism. In fact, variations of the VEGF gene (rs2146323 and rs3025039) have only been associated with an increased risk of GDM, and therefore could be caused by an abnormal expression of VEGF, increasing its levels in these patients (Dong, 2019).

However, molecular mechanisms explaining this dysfunction have not been described for these polymorphisms. Evidence for rs735286 indicates that it is located in the second VEGF intron, and this variant involves a region that has putative binding sites for transcription factors such as the myeloid zinc finger protein (MZF-1), that regulates *VEGFA* expression. Therefore, rs735286 could affect the transcription process and cause changes in splicing (Morris et al., 1994; Churchill et al., 2008).

Although the rs2146323 polymorphism is also found in intron 2, it is located relatively distant from these binding sites, so the mechanism would be apparently different (Churchill et al., 2008). On the other hand, it has been suggested that rs3025039, being in a 3′-UTR region, could alter the conformation of the mRNA, which would decrease the transcript degradation, causing an increase in VEGF expression (Dibbens et al., 1999; Tahara et al., 2009).

#### 2.5.2 *MIF* Genetic Variant Associated to GDM

The macrophage migration inhibitory factor (MIF), is a pro-inflammatory cytokine secreted by T-lymphocytes in response to delayed-type hypersensitivity, which exerts an inhibitory effect on macrophage migration. The rs755622 polymorphism of this gene (MIF-173G/C) is associated with higher GDM risk (OR: 1.59) (Li et al., 2016), and the rs1007888 polymorphism is related to high levels of blood glucose and insulin (Zhan et al., 2015).

MIF is a pro-inflammatory cytokine that is secreted in response to delayed-type hypersensitivity and exerts an inhibitory effect on macrophage migration (Hamidi et al., 2019). It has been described that MIF controls underlying metabolic and inflammatory processes during periods of stress, regulating glucose homeostasis and macrophage infiltration into adipose tissue. Also, MIF is expressed and secreted by adipose tissue, evidencing higher levels in obesity (Mejia-Montilla et al., 2015).

The rs755622 variation is located in the promoter region of *MIF* and the C allele is related with a higher transcriptional activity of the *MIF* gene. In fact, patients with T2DM have shown increased MIF levels (Hamidi et al., 2019). Also, it has a direct association with insulin resistance through the production of some pro-inflammatory cytokines and adipokines, including resistin and IL-6. Additionally, this variation has been related to an increased risk of GDM in Chinese women; hence, more studies should be carried out in other populations to determine the role of this polymorphism in this pregnancy disease (Li et al., 2016). However, more studies are needed to confirm the association of these variants and GDM.

### 2.6 Other SNPs Associated With GDM

The IRS-1 is an intracellular adaptor protein that plays a key role in insulin signaling. The *IRS1* rs1801278 (C > T) variant has been related to GDM in meta-analyses (Zhang Y. et al., 2014; Wu et al., 2016), similarly to the rs7578326 (A > G) polymorphism (Voight et al., 2010; Zheng et al., 2013; Zhao et al., 2017). *IRS1* rs1801278 is a missense polymorphism that decreases the phosphorylation of IRS-1 *in vitro* (McGettrick et al., 2005), reducing the binding of p85 to IRS-1 and the activity of PI3K in different cell lines (Hribal et al., 2000; Sentinelli et al., 2006), and leading to insulin resistance. On the other hand, rs7578326 is an intron variant positioned in a distal regulatory element that targets *IRS1* (Lu et al., 2013). The risk allele A removes a CpG site and avoids its methylation (Dayeh et al., 2013). This genetic variant has been linked to higher levels of transcript in skeletal muscle, with no changes in the levels of IRS1 mRNA (Soyal et al., 2015). Further studies are needed to elucidate the exact role of this SNP on the pathophysiology of T2DM and GDM.

The SNP 43, SNP 44, SNP 63, and Indel 19 variants of the *CAPN10* gene were not associated with GDM risk (Shaat et al., 2005; Neuhaus et al., 2013; Khan et al., 2014; Zhang et al., 2019; Ustianowski et al., 2021), except in some genetic association models of SNP 63 and SNP 44 (Cui et al., 2016), and in *CAPN10* SNP-43/19/63 haplotypes (Hou et al., 2017). Nevertheless, the Indel-19 and SNP-19 variants were associated with higher glucose levels in GDM women (Castro-Martínez et al., 2018). In addition, GWAS have discovered several genetic variants associated with both T2DM and GDM. Among them, the rs7754840 polymorphism of the *CDKAL1* gene (C-allele) and the rs10830962 polymorphism of the *MTNR1B* gene (G-allele) have been associated to a higher GDM risk (OR: 1.518; OR: 1.454, respectively). The risk alleles of these polymorphisms have also been associated with a decreased fasting insulinemia in GDM women (Kwak et al., 2012).

In a meta-analysis, other polymorphisms were associated with the risk of developing GDM, such as the A allele of the rs1800629 polymorphism in the TNF-α gene (OR 2.69), the T-allele of the rs4402960 SNP in the *IGF2BP2* gene (OR: 1.22), and the G-allele of the rs10830963 variant in the *MTNR1B* gene (OR: 1.28). This last variant is also associated with GDM risk, in Asian (OR 1.23) and Caucasian (OR: 1.49) populations, and women with pre-gestational BMI ≥25 kg/m^2^ (OR 1.24) (Wu et al., 2016).

## 3 Concluding Remarks

**FIGURE 1:**
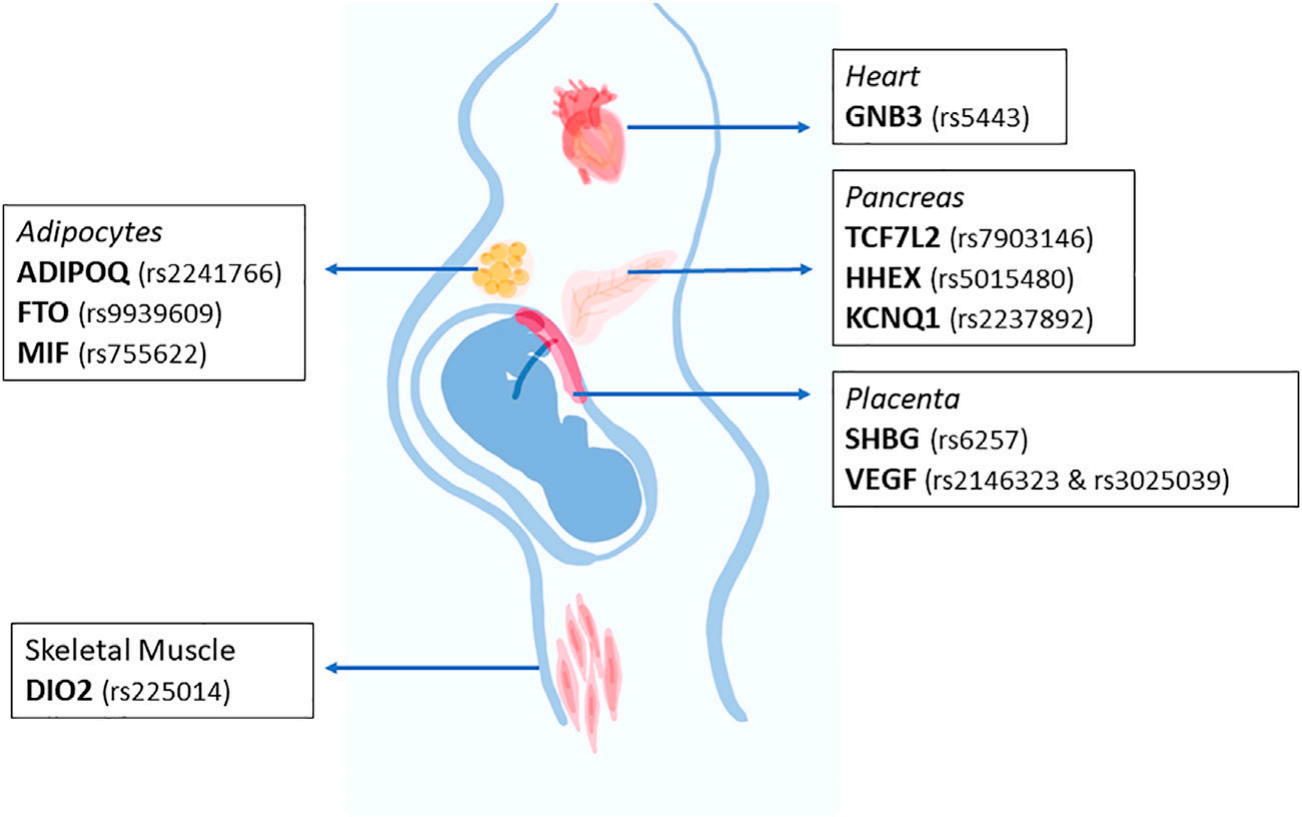
Major organs affected by the main SNPs associated with gestational diabetes mellitus. The main organs affected by the SNPs described in this review are heart, adipose tissue, pancreas, and skeletal muscle. Interestingly, the SNPs described would affect some functions in the indicated organ, favoring the onset of gestational diabetes mellitus.

Scientific literature has evidenced various polymorphisms associated with GDM, which can affect one or several functions. However, the specific mechanism by how these polymorphisms can affect the body physiology has not been addressed. Figure 1 summarizes the main tissues that would be affected by the described polymorphisms during pregnancy. We emphasize that the main organs associated with energy metabolism such as skeletal muscle, adipose tissue and pancreas could be involved, but clearly the placenta is an organ that plays an important role in the context of a pregnancy-related pathology. Although the placenta is a fetal organ that is genetically different from the mother, it is difficult to know whether maternal genetic alterations could be linked to placental defects.

**FIGURE 2:**
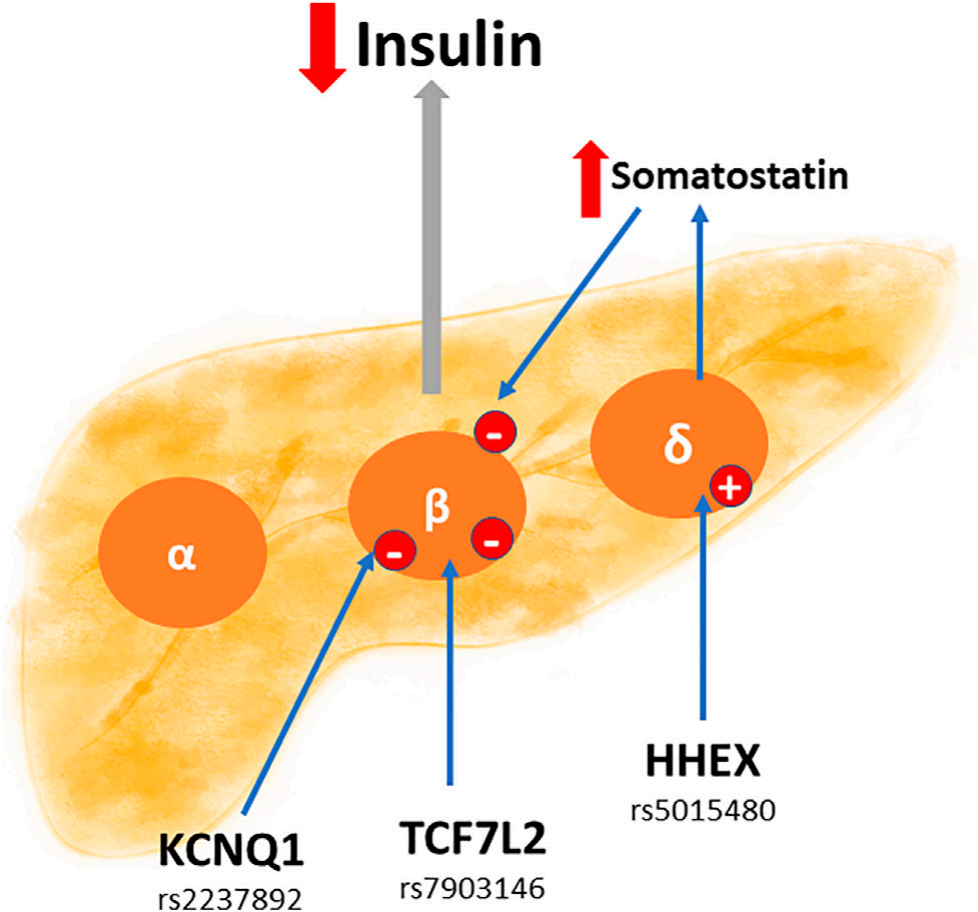
Potential SNPs capable of reducing the production of maternal insulin at a pancreatic level. Some SNPs can affect the secreting function of pancreatic insulin. In this sense, the SNPs *KCNQ1* rs2237892, *TCF7L2* rs7903146 and *HHEX* rs5015480, reduce (down red arrow) direct or indirectly the production and secretion of insulin at the level of the beta cells (β). Moreover, the same *HHEX* SNP can stimulate (up red arrow) somatostatin secretion in delta cells (δ), and this hormone is an inhibitor of insulin secretion. Impaired insulin production favors insulin resistance, and therefore the appearance of gestational diabetes mellitus.

**FIGURE 3:**
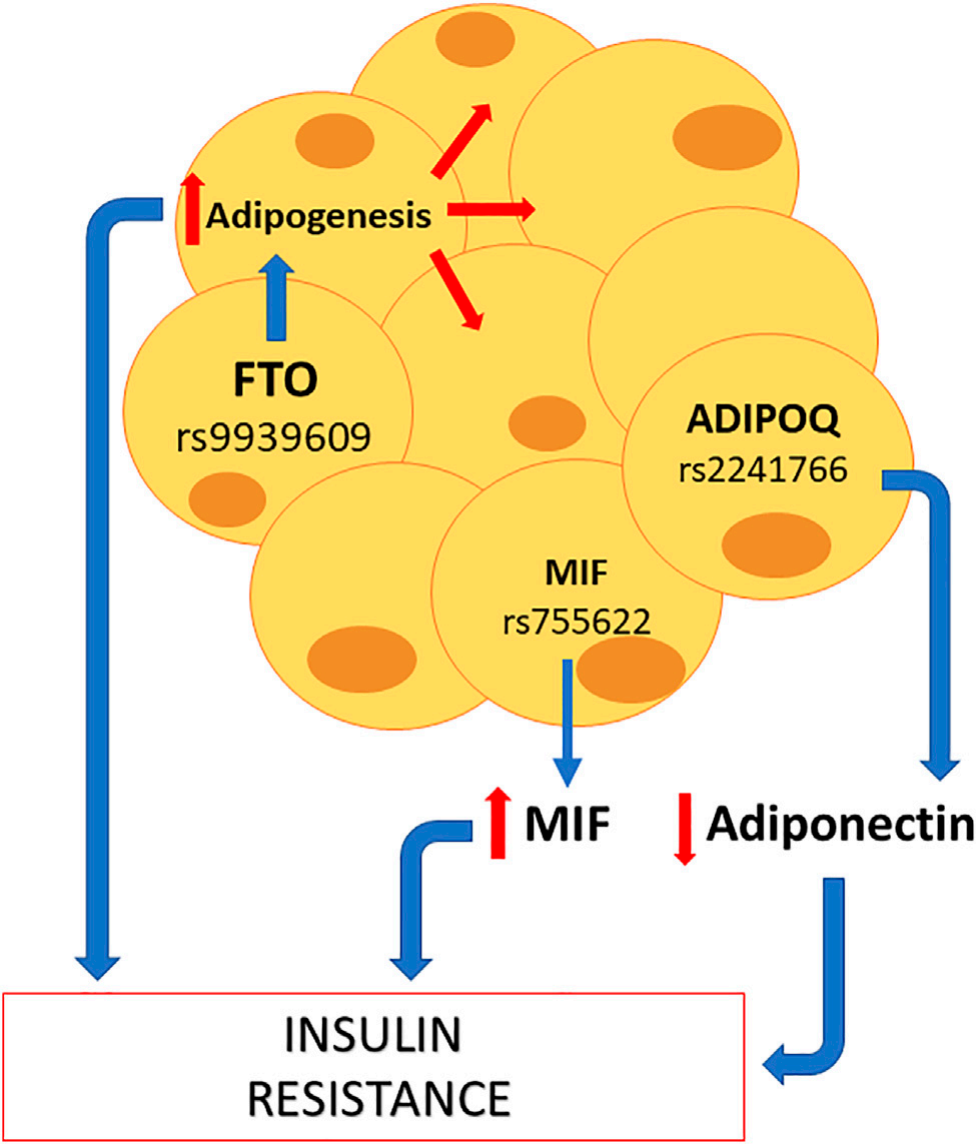
Potential SNPs capable of promoting maternal insulin resistance at adipose level. Some SNPs that can affect the adipocyte function are *FTO* rs9939609, which increases adipogenesis; *MIF* rs755622, which increases MIF levels; and *ADIPOQ* rs2241766, which reduces adiponectin levels. All these three effects favor the appearance of insulin resistance, and subsequently, gestational diabetes mellitus.

The endocrine pancreas is a key organ in the synthesis of several hormones such as somatostatin, glucagon, and insulin. The latter is relevant to understand the pathophysiology of GDM. In fact, as mentioned earlier, GDM courses with supraphysiological insulin resistance. Accordingly, and as shown in Figure 2, the polymorphisms *KCNQ1* rs2237892, *TCF7L2* rs7903146, and *HHEX* rs5015480 are closely linked in the reduction of insulin secretion by beta cells, which could explain GDM development. One cause of insulin resistance is obesity. That explains why polymorphisms that have a consequence on metabolism, such as adipose tissue hyperplasia, are associated with GDM, as shown in Figure 3. Indeed, the polymorphisms *FTO* rs9939609, *ADIPOQ* rs2241766, and *MIF* rs755622 are strongly linked to the presence of insulin resistance.

Unfortunately, evidence is insufficient to understand all the pathophysiological changes observed in GDM at the genetic level. In fact, GDM, as well as T2DM, are polygenic pathologies. However, current scientific evidence leads us to believe that certain polymorphisms could favor alterations in key organs, such as the pancreas and the adipose tissue, promoting insulin resistance during pregnancy and GDM.
